# Influence of lifting straps on force, rate of force development and impulse outputs during isometric mid-thigh pull: Evidence from judo and resistance-trained populations

**DOI:** 10.1371/journal.pone.0357488

**Published:** 2026-09-03

**Authors:** Hung-Chih Yeh, Chia-An Ho, En-Yu Chang, Hei-Tung Lau, Chih-Wen Hsu, Hsin-Yu Tu, Pei-Hsuan Wang, Chin-Shan Ho

**Affiliations:** 1 Sports Education Center, National Ilan University, Ilan, Taiwan; 2 Graduate Institute of Sports Science, National Taiwan Sport University, Taoyuan, Taiwan; Sheffield Hallam University, UNITED KINGDOM OF GREAT BRITAIN AND NORTHERN IRELAND

## Abstract

The isometric mid-thigh pull (IMTP) is a reliable method for assessing whole-body maximal isometric strength; however, the influence of lifting straps on IMTP performance remains unclear, particularly in sports such as judo that rely heavily on grip strength. This study recruited 30 male participants, including 15 judo athletes and 15 recreationally resistance-trained undergraduates, who completed IMTP tests under strapped and non-strapped conditions in a randomized order. Peak force (PF), rate of force development (RFD), and impulse (IP) were measured using a 1000 Hz force plate, and a two-way mixed ANOVA was used to examine the effects of group and strap condition. The results showed no significant interaction between group and condition. However, significant main effects of both group and strap condition were observed for most variables (*p* < 0.05), with higher PF, RFD, and IP recorded under the strapped condition. Judo athletes also demonstrated greater grip strength (51.3 ± 3.4 kg) than the resistance-trained group (44.7 ± 3.3 kg, *p* < 0.01). These findings suggest that the use of lifting straps enhances IMTP performance and may reduce the influence of grip strength limitations on maximal force expression. Therefore, when the purpose of IMTP testing is to assess maximal force production, the use of lifting straps may be considered to minimize the potential influence of grip-related limitations and to standardize testing procedures across athletes.

## Introduction

Judo is a combat sport in which athletes aim to gain advantages by throwing their opponents or controlling them on the ground. To consistently execute technical and tactical skills under high-intensity competition conditions, athletes must possess superior physical fitness [1–3]. Performance in judo competitions not only depends on technical and tactical proficiency but is also highly reliant on maximal strength, muscular endurance, and rate of force development. According to a systematic review, judo athletes undergoing resistance training can significantly improve their maximal dynamic and isometric strength, as well as throwing performance, body stability, and sport-specific physical fitness [4]. Furthermore, as a grappling-type combat sport, judo frequently involves performing techniques under high muscular loading against an opponent’s resistance, such as gripping or immobilizing [5–7].

Traditional one-repetition maximum (1RM) testing often results in fatigue, increases the risk of injury, and provides limited information, focusing solely on the maximum load lifted [8]. To address these limitations, the isometric mid-thigh pull (IMTP) developed in the 1990s has gained popularity as a safe, efficient method to assess isometric strength [9]. IMTP replicates the second pull phase of the clean, with the bar fixed at mid-thigh height, requiring athletes to exert maximal force under static, joint-stable conditions, minimizing dynamic load risk [10,11]. As a multi-joint test, it provides force-time metrics such as peak force and rate of force development (RFD) [12]. IMTP outcomes show moderate to strong correlations with 1RM values in lifts like the clean, jerk, squat, and deadlift (*r* = 0.64–0.97), and are also linked to performances in jumping, sprinting, and change of direction tasks [13–15]. Therefore, IMTP may serve as a practical tool for strength assessment and training monitoring in judo athletes, while also providing complementary information related to 1RM performance.

Lifting straps are widely utilized in resistance training, primarily aiming to transfer part of the load from the fingers to the wrists, thereby allowing the legs and back to more effectively transfer force to the barbell [16,17]. Given that insufficient grip strength can potentially limit maximal force output during IMTP tests and compromise the accuracy of the results [8]. Previous IMTP-related studies typically included lifting straps as a standard procedure. For instance, participants in past research have included college athletes with over two years of structured strength training [18], collegiate rowers and soccer players [19], weightlifters [20,21] and rugby players [15]. However, grip strength plays a particularly decisive role in judo performance, as athletes must continuously establish and maintain gripping control over their opponents during both offensive and defensive actions. Previous studies have identified handgrip strength as a key physical characteristic in grappling combat sports, including judo, Brazilian jiu-jitsu, and wrestling [5,22,23].

Previous studies have examined the use of lifting straps in different populations and have shown that lifting straps may improve pulling-related performance. For example, female athletes were found to benefit from lifting strap use during deadlift performance [24]. In addition, studies in men who regularly engaged in strength training have reported that lifting straps may enhance pulling performance in movement patterns such as the deadlift and IMTP [25]. Taken together, no study to date has specifically investigated whether the use of lifting straps affects IMTP performance in judo athletes. Given the sport-specific characteristics of judo, which impose substantial demands on grip strength, judo athletes may respond differently to strapped and strapless IMTP conditions compared with individuals with weight-training experience. Therefore, the present study aimed to examine whether the presence or absence of lifting straps differentially affects IMTP performance across groups. Because judo athletes typically have greater sport-specific grip demands than the weight-training experience group, we hypothesized that their IMTP performance would not differ significantly between the strapped and strapless conditions.

### Materials and methods

#### Subjects

G*Power 3.1.9.7 was used to calculate the appropriate sample size for this study. Peak force (PF) was selected as the primary outcome variable because it is the most commonly reported and widely accepted indicator of maximal force production during IMTP testing. Using a statistical power of 0.80, an α level of 0.05, and an assumed effect size (Cohen’s f = 0.27), the required total sample size was determined to be 30 participants, which satisfied the power criterion. [26]. Thirty male college students voluntarily participated in this experiment, including 15 first-level judo black belt athletes (Judo group) (age 22.06 ± 2.04 years, body mass 74.29 ± 10.38 kg, height 172.73 ± 5.65 cm), and 15 undergraduates with at least 6 months of weight-training experience were recruited for the resistance-trained group (RT group) (age: 20.06 ± 2.26 years; body mass: 77.93 ± 10.03 kg; height: 174.1 ± 5.15 cm). The ≥ 6-month criterion was defined according to the criteria used in previous studies [20]. Participants with any contraindications to exercise or any recent upper or lower limb injuries within the last six months that could potentially impede their safe completion of the testing procedures were excluded. Each participant was required to attend one laboratory session for testing, during which they were briefed on the overall experimental procedures and completed approximately 1 hour of testing (including warm-up and familiarization with the procedures) in a laboratory environment set at an average temperature of 23°C. Participants failing during testing or training (e.g., due to injury during training or any other factor) had their personal information and data excluded. All participants provided written informed consent, and the study was approved by the Institutional Review Board of Fu Jen Catholic University (C111175). Participant recruitment was conducted from June 26 to October 11, 2023.

Participants performed two IMTP tests (with and without lifting straps) on the same day and at the same time. Both participant groups completed the strap and no-strap conditions in a randomized and counterbalanced order to evaluate the effects of different populations and lifting strap usage on IMTP performance. IMTP outcome included peak force (PF), peak force at 250 ms (PF_250_), rate of force development (RFD), RFD at 250 ms (RFD_250_), impulse over 1s (IP_1_) and impulse over 5s (IP_5_).

All participants engaged in a standardized warm-up comprising 10 bodyweight squats and 10 bodyweight lunges, followed by two 5-second IMTP trials performed at 50% and 75% of maximal effort, with a 2-minute rest interval between each exercise. [27,28].

IMTP testing was conducted on a force plate (BMS400600, OPTIMA, USA) at a sampling rate of 1000 Hz, utilizing the IMTP test kit (IMTP Rack, Kairos Strength, USA) [29]. To accommodate athletes of varying body sizes, the barbell height was adjustable in 2 cm increments based on individual needs. Participants performed IMTP tests under both lifting strap and no-strap conditions, with the hands securely fastened to the barbell using lifting straps during the strap condition. In all trials, the knee joint angle was standardized between 125° and 145°, while the hip joint angle ranged from 140° to 150° [30]. Prior to each test, participants were carefully positioned according to the predetermined joint angles, which were verified using a goniometer to ensure consistency and accuracy across all testing sessions.

Participants completed four IMTP trials in total, with a 2-minute rest interval between each trial. The strapped and non-strapped IMTP conditions were administered in a randomized order. After stepping onto the force plate, participants adjusted their hand spacing on the bar to a self-selected comfortable width and used a pronated grip throughout the test. They then wrapped the lifting straps around their hands and assumed the ready position. The lifting straps were made of rubber material and were approximately 5 cm in width. The strap length was individually adjusted to ensure participant comfort while allowing a secure grip on the barbell. The tester initiated the test with a countdown of “3, 2, 1. pull,” prompting participants to pull the barbell with maximal effort while pushing their feet down into the force plate. Each trial lasted 5 seconds, during which strong verbal encouragement was provided, followed by a 2-minute rest interval between trials [18,31,32]. The tester ensured data availability for each trial, and if countermovement, excessive pre-tension, or leaning on the bar prior to the pull occurred, participants were instructed to rest for 2-minutes before repeating the trial. Participants performed two maximal effort tests under different movement specifications, ensuring an upright torso with the barbell positioned at mid-thigh (midpoint between the center of the patella and the iliac crest) [8]. Following previous recommendations, if the difference between the two trials exceeded 250N, a third trial was conducted [33]. The mean of the two trials was used for statistical analyses [33,34]. The main IMTP variables, including PF, PF250, RFD, RFD250, IP1, and IP5, were calculated using MATLAB. To account for differences in body size, all force-related variables were normalized to body mass and expressed relative to body weight. Force–time curves were digitally filtered using a second-order Butterworth low-pass filter with a cutoff frequency of 10 Hz. [20]. The onset of pulling was identified as the point at which the vertical ground reaction force differed by more than 40 N from the average body weight recorded during the weighing phase [35].

MIHS was assessed using a Takei 5401 digital handgrip dynamometer (Takei Scientific Instruments Co., Ltd., Tokyo, Japan). The MIHS assessment was conducted following a standardized protocol to ensure consistency and reliability. Participants performed the test with both hands. Before testing, the dynamometer was adjusted to fit each participant’s hand size to ensure an optimal grip span. During the test, participants stood in a natural position with their shoulders adducted, holding the dynamometer without body contact, while wrist flexion was not allowed to maintain standardized testing conditions. Each participant performed three trials, with the best result recorded for analysis. The MIHS test required participants to perform a maximal voluntary contraction for 5 seconds in each trial [36].

### Statistical analysis

All statistical analyses were conducted using SPSS software (IBM Corp., New York, NY, USA). Prior to the main analyses, normality was assessed using the Shapiro–Wilk test and homogeneity of variance was evaluated using Levene’s test. The majority of variables met the assumption of normality, and all variables satisfied the assumption of homogeneity of variance. To evaluate the effects of lifting strap conditions on IMTP outcomes, a two-way mixed-design ANOVA was employed. Mauchly’s test of sphericity was used to assess the assumption of sphericity, and if violated, the Greenhouse–Geisser correction was applied. Bonferroni-adjusted post hoc comparisons were conducted only for variables showing a significant Group × Strap Condition interaction to examine simple main effects. An independent t-test was used to compare grip strength between the two groups. Effect sizes were calculated using partial eta squared (η²) and interpreted as small (0.01), medium (0.06), and large (0.14) for both main and interaction effects. [37]. Statistical significance was set at *α* < 0.05, and data were reported as mean ± standard deviation (SD).

## Results

MIHS differed significantly between the judo group and the RT group for both the dominant and non-dominant hands. For the dominant hand, MIHS was significantly higher in the judo group than in the RT group (51.31 ± 3.39 vs. 44.65 ± 3.32 kg, *p* < 0.01). Similarly, for the non-dominant hand, MIHS was also significantly higher in the judo group than in the RT group (43.96 ± 4.99 vs. 39.92 ± 3.99 kg, *p* = 0.02).

Table 1 presents all IMTP parameters, and none of the parameters demonstrated a significant interaction effect.

**Table 1:** Two-way ANOVA results for IMTP outcomes across different condition (with or without lifting straps).

Parameters	*F*	Condition effect *p* value	η²	*F*	Group effect *p* value	η²	*F*	Condition x group *p* value	η²
PF (N/BW)	16.04	<0.01	0.36	42.63	<0.01	0.60	0.25	0.61	0.00
PF_250_ (N/BW)	14.75	<0.01	0.34	16.09	<0.01	0.36	0.10	0.75	0.00
RFD (N/s/BW)	21.7	<0.01	0.43	5.38	0.02	0.16	0.01	0.89	0.00
RFD_250_ (N/s/BW)	6.50	0.01	0.18	14.96	<0.01	0.34	1.46	0.23	0.05
IP_1_ (N*s/BW)	4.53	0.04	0.13	12.78	0.01	0.31	1.65	0.20	0.05
IP_5_ (N*s/BW)	3.46	0.07	0.11	39.90	<0.01	0.58	0.41	0.52	0.01

PF: Peak Force; RFD: Rate of force development; Force_250_: Force at 250 ms; RFD_250_: RFD 0–250 ms; IP_1_: impulse over one second; IP_5_: impulse over five second; BW: body weight.

As shown in Table 2, significant differences were observed for all IMTP outcomes except RFD under the strapped condition and IP1 under the strapless condition.

**Table 2:** Group differences in IMTP measures with and without Lifting Straps.

parameter	Condition	Judo	RT	*p* value
PF (N/BW)	Straps	4.01 ± 0.81	2.6 ± 0.65	<0.01
	No Straps	3.30 ± 0.77	2.05 ± 0.53	<0.01
PF_250_ (N/BW)	Straps	3.08 ± 0.85	2.25 ± 0.55	<0.01
	No Straps	2.36 ± 0.84	1.64 ± 0.49	<0.01
RFD (N/s/BW)	Straps	25.79 ± 5.82	21.76 ± 7.07	0.10
	No Straps	19.81 ± 7.24	15.44 ± 3.74	0.04
RFD_250_ (N/s/BW)	Straps	11.50 ± 4.86	7.22 ± 1.94	0.01
	No Straps	8.46 ± 3.26	6.13 ± 1.86	0.02
IP_1_ (N*s/BW)	Straps	2.74 ± 0.73	1.80 ± 0.45	<0.01
	No Straps	2.11 ± 1.09	1.65 ± 0.46	0.14
IP_5_ (N*s/BW)	Straps	14.09 ± 3.52	8.54 ± 2.53	<0.01
	No Straps	11.97 ± 4.06	7.52 ± 2.21	<0.01

PF: Peak Force; RFD: Rate of force development; Force_250_: Force at 250 ms; RFD_250_: RFD 0–250 ms; IP_1_: impulse over one second; IP_5_: impulse over five second; BW: body weight.

## Discussion

This study aimed to investigate the effects of lifting straps on IMTP performance in judo athletes and undergraduates with at least six months of weight-training experience. The results did not support our hypothesis, as no significant interaction effects between group and strap condition were observed for any of the measured variables. However, significant main effects of group and strap condition were observed for most parameters. These findings indicate that, regardless of strap condition, judo athletes demonstrated superior overall IMTP performance compared with the weight-training experience group.

The judogi (judo uniform) in judo is not only a basic piece of equipment but also a central tool for technical execution and tactical application. Athletes must grip the judogi to establish control and initiate attacks, with the type of grip directly influencing the choice and effectiveness of techniques. Furthermore, scoring techniques must be executed with contact to the judogi, highlighting its importance in both competition rules and practical performance [5]. Therefore, grip strength has become a crucial factor influencing judo performance, especially under conditions requiring isometric strength endurance and maximal isometric strength [38]. This emphasis has made grip strength training one of the essential components in judo athletes’ regular training routines. Due to long-term, sport-specific training, judo athletes tend to exhibit superior maximal MIHS compared to non-athletes. Previous research aligns with the findings of the present study. For instance, the results of Honorato et al. [39] also demonstrated that judo athletes exhibited superior grip strength compared to non-athletes.

In the present study, the findings under the strapped condition were consistent with previous studies, suggesting that lifting straps can provide benefits for pulling-related movements and enhance IMTP performance [24,25]. This may be because lifting straps reduce the limiting effect of grip strength during the IMTP, allowing force to be transmitted to the bar more effectively and thereby enhancing overall pulling performance. Although judo athletes demonstrated superior overall IMTP performance compared with the resistance-trained group, the absence of a significant Group × Strap Condition interaction suggests that the influence of lifting straps was generally consistent across both groups. Therefore, the benefits associated with lifting strap use during IMTP testing appear to be independent of training background and may reflect a general enhancement in force transmission rather than a sport-specific effect.

Although IMTP testing provides coaches with a safe and efficient method for estimating an athlete’s 1RM and developing individualized resistance-training programs [11,30], the present findings suggest that relying solely on data obtained under the strapless condition may not fully reflect actual IMTP performance. Therefore, even for judo athletes, the use of lifting straps during IMTP assessment may still be necessary to obtain a more complete evaluation of performance. This study presents several limitations that should be considered when interpreting the findings. First, the relatively small and homogeneous sample may have limited statistical power, particularly for detecting interaction effects, and reduced the generalizability of the findings. Second, although the IMTP is a validated measure of maximal isometric force, it may not capture all aspects of dynamic performance relevant to judo, such as explosive throwing or rapid directional changes. Third, only one testing session was conducted for both conditions, which may not account for day-to-day variability in strength performance. Fourth, the present study lacked key background information, such as training years, training frequency, typical training volume, and overall training background. Therefore, the findings should be interpreted with caution, particularly when comparing the characteristics of the participant groups and generalizing the results. Lastly, the lack of direct physiological or electromyographic measurements limits insights into the underlying neuromuscular mechanisms affected by strap usage. Future studies should consider including a more diverse sample, longitudinal training data, and neuromuscular assessments to enhance the understanding of lifting strap effects in combat sports contexts.

## Conclusions

In summary, this study investigated the influence of lifting straps on IMTP performance in judo athletes and recreationally trained undergraduates. Although no significant interaction between group and strap condition was observed, the use of lifting straps significantly enhanced IMTP performance across both groups. These findings suggest that grip strength limitations may influence maximal force expression during IMTP testing. Therefore, practitioners should be aware that the use of lifting straps may affect IMTP outcomes and should consider standardizing testing conditions when assessing and monitoring strength performance. From a practical perspective, lifting straps may help reduce the influence of grip-related limitations and provide a more consistent evaluation of maximal force production across different athletic populations.

## Supporting information

S1 Data Raw data. (XLSX)
